# Ultra-slim pancreatoscopy-guided laser lithotripsy for retrieval of a kinked guidewire caused by a pancreatic duct stone

**DOI:** 10.1055/a-2849-6026

**Published:** 2026-04-24

**Authors:** Noriyuki Hirakawa, Kazumasa Nagai, Ryosuke Tonozuka, Shuntaro Mukai, Kyoko Asano, Takao Itoi

**Affiliations:** 1 Department of Gastroenterology and Hepatology 13112 Tokyo Medical UniversityTokyoJapan

Pancreatic duct stones are a common complication of chronic pancreatitis, and various therapeutic approaches are available when symptoms occur 1 2 3 . Endoscopic treatment of pancreatic duct stones is often technically challenging 2 . However, laser lithotripsy has been reported to have favorable clinical outcomes in the treatment of these stones 4 . In addition, the usefulness of an ultra-slim peroral pancreatoscope (POPS; Briview CHV-US120J-U, 7.8 Fr; Shanghai SeeGen Photoelectric Technology Co., Ltd, Shanghai, China) has recently been reported 5 . This report describes a case in which a kinked guidewire caused by a pancreatic duct stone at a stricture site was successfully retrieved by ultra-slim pancreatoscopy-guided laser lithotripsy.

A 52-year-old woman had recurrent episodes of pancreatitis caused by pancreatic duct stones. She had previously undergone extracorporeal shock wave lithotripsy and endoscopic pancreatic sphincterotomy. She was admitted to our hospital for the endoscopic removal of pancreatic duct stones. The pancreatic duct was cannulated, and pancreatography revealed pancreatic duct stones and strictures in both the pancreatic head and tail. Stone extraction was attempted using a balloon catheter and basket mechanical lithotripsy. During the procedure, the guidewire encountered a stone and became kinked at a pancreatic duct stricture in the tail ( Fig. 1 ). Dilation of the stricture was attempted using a drill dilator; however, the guidewire could not be retrieved. Therefore, an additional guidewire was placed in the pancreatic duct. Given the presence of a stricture in the pancreatic duct in the head, an ultra-slim POPS was advanced over the guidewire ( Fig. 2 **a** ). Under pancreatoscopic visualization, the pancreatic stone and the kinked guidewire were identified. The pancreatic stone was directly visualized and fragmented using laser lithotripsy ( Fig. 2 **b** ). Next, the kinked guidewire was successfully retrieved without fracture. Finally, endoscopic nasopancreatic drainage was performed, and the procedure was completed. No procedure-related adverse events were observed ( Video 1 ).

**Fig. 1:**
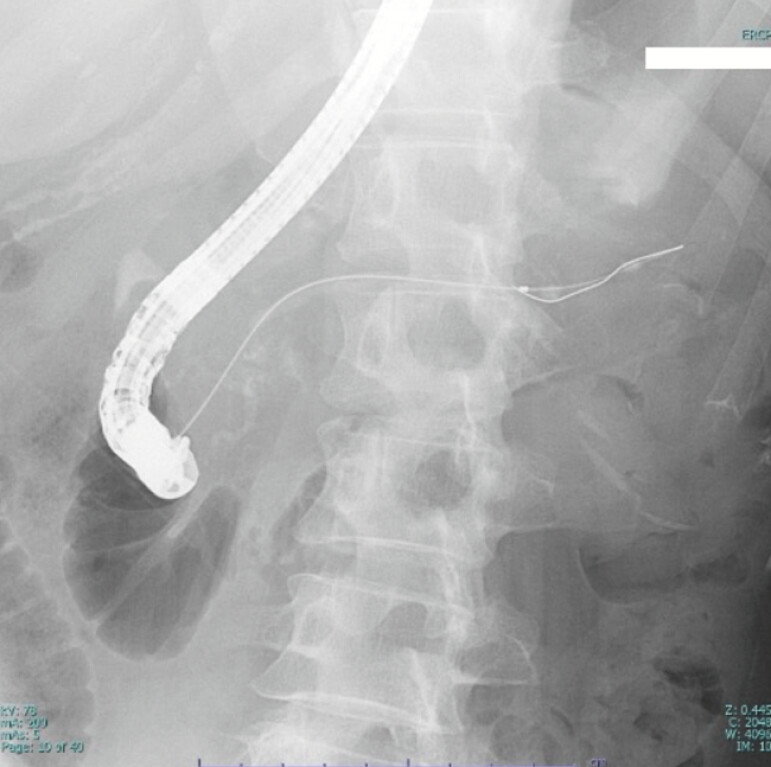
A radiograph showing the kinked guidewire in the pancreatic duct.

**Fig. 2:**
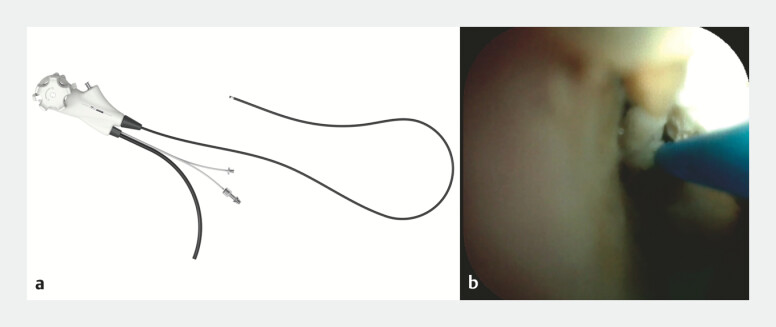
**a** An ultra-slim peroral pancreatoscope with an effective length of 2,150 mm, an outer diameter of 7.8 Fr, and a 0.9-mm working channel. **b** Pancreatoscopy-guided laser lithotripsy for pancreatic duct stones.

Retrieval of a kinked guidewire using ultra-slim pancreatoscopy-guided laser lithotripsy for a pancreatic duct stone.Video 1

Endoscopy_UCTN_Code_TTT_1AR_2A

## References

[1] Dumonceau JM Delhaye M Tringali A Endoscopic treatment of chronic pancreatitis: European society of gastrointestinal endoscopy (ESGE) clinical guideline Endoscopy 2012 44 784 800 10.1055/s-0032-1309840 22752888

[2] Tandan M Nageshwar Reddy D Endotherapy in chronic pancreatitis World J Gastroenterol 2013 19 6156 6164 10.3748/wjg.v19.i37.6156 24115811 PMC3787344

[3] Cahen DL Gouma DJ Laramée P Long-term outcomes of endoscopic vs surgical drainage of the pancreatic duct in patients with chronic pancreatitis Gastroenterology 2011 141 1690 1695 21843494 10.1053/j.gastro.2011.07.049

[4] Maydeo A Kwek BE Bhandari S Single-operator cholangioscopy-guided laser lithotripsy in patients with difficult biliary and pancreatic ductal stones (with videos) Gastrointest Endosc 2011 74 1308 1314 22136776 10.1016/j.gie.2011.08.047

[5] Kanazawa Y Nagai K Hirakawa N Clinical application of ultra-slim peroral cholangioscopy for a giant common bile duct stone: An initial experience J Hepatobiliary Pancreat Sci 2025 33 10.1002/jhbp.70033 41362257

